# Pathological Complete Response to Neoadjuvant Chemotherapy in Patients With Breast Cancer: The Relationship Between Inflammatory Biomarkers and Molecular Subtypes

**DOI:** 10.7759/cureus.14774

**Published:** 2021-04-30

**Authors:** Kübra Kaytaz Tekyol, Gunay Gurleyik, Ayşegül Aktaş, Fugen Aker, Eda Tanrikulu, Davut Tekyol

**Affiliations:** 1 General Surgery, Kovancılar State Hospital, Elazığ, TUR; 2 General Surgery, University of Health Sciences, Haydarpaşa Numune Training and Research Hospital, İstanbul, TUR; 3 Pathology, University of Health Sciences, Haydarpaşa Numune Training and Research Hospital, İstanbul, TUR; 4 Oncology, University of Health Sciences, Haydarpaşa Numune Training and Research Hospital, İstanbul, TUR; 5 Emergency Medicine, University of Health Sciences, Haydarpaşa Numune Training and Research Hospital, İstanbul, TUR

**Keywords:** breast cancer, chemotherapy, plr, nlr, pcr, luminal, triple negative, her-2

## Abstract

Purpose

Among patients with breast cancer, pathological complete response (pCR) to neoadjuvant chemotherapy (NAC) is an important prognostic predictor of survival. This study aimed to investigate the relationship between platelet-lymphocyte ratio (PLR) and neutrophil-lymphocyte ratio (NLR) along with overall pCR.

Method

A total of 150 patients with breast cancer who were first administered NAC and then operated on were retrospectively evaluated. Neutrophil-lymphocyte ratio and PLR obtained from the complete blood count analysis performed immediately before NAC treatment were analyzed. The cut-off value was calculated as 150 for PLR and 2.24 for NLR. We studied the predictive value of NLR and PLR levels for the pathologic response of breast cancer to NAC.

Results

Pathological complete response was observed in 34.7% (n = 52) of the patients, pCR in the breast in 42.7% (n = 64), and that in the axilla in 44% (n = 66). There was a statistically significant difference between the pCR rates according to the PLR levels (p = 0.013). In addition, a statistically significant difference was found in the pCR rates in the breast and axilla according to PLR levels (p = 0.018, p = 0.009). Patients with low PLR in the human epidermal growth factor receptor 2 (HER-2) group had significantly higher axillary pCR rates than in those with high PLR (p = 0.019).

Conclusions

A low PLR level showed high chemotherapy sensitivity independent of molecular subtypes in the treatment of breast cancer with NAC. The PLR level can serve as a predictive marker of the therapeutic effect of NAC on the breast and axilla. Low PLR levels in HER-2 enriched groups can predict pCR in the axilla.

## Introduction

In recent years, the use of neoadjuvant chemotherapy (NAC) has increased in the treatment of breast cancer. The risk of both locoregional and distant metastases is also reduced with subtypes-specific systemic treatments performed before surgery. On the other hand, NAC provides de-escalating surgery in the breast and the axilla that pathological complete response (pCR) is achieved in some patients. Pathological complete response is an effective factor in predicting overall survival and disease-free survival which are generally improved (increased) after complete response to chemotherapy. Different features of tumors, especially molecular subtypes information are used to predict the response to NAC. Additionally, some inflammatory biomarkers are also studied for their effects on pCR prediction [1-4]. Neutrophils play an important role in immune response since they affect both innate immunity and cell signaling in the adaptive immune response, and they are also a major factor in the suppression of cancer progression. Inflammation that begins with the passage of neutrophils from the circulation to tissues is an essential element not only for infections but also for immune response in cancer. Neutrophils act on the elements of the complement system and the adaptive immune system, thereby strengthening the inflammatory response [5-8]. Platelets can also be involved in the inflammatory response by increasing angiogenesis or by providing growth factor release. Substances released from platelets are required for the activation of endothelial cells surrounding the vascular tract in inflammation. The presence of different subtype T lymphocytes in the tumor microenvironment (tumor-infiltrating lymphocytes) has also been associated with a good prognosis in various malignancies [6]. Lastly, neutrophil-lymphocyte ratio (NLR) and platelet-lymphocyte ratio (PLR) are inflammatory markers that affect total survival in many malignancies. They are reported to be predictive of chemo-sensitivity. The NLR and PLR values ​​are also used as prognostic and survival markers in many diseases other than malignancy [9-11]. 

The aim of this study was to investigate the role of NLR and PLR in predicting pCR in women with breast cancer and their relationship with molecular subtypes.

## Materials and methods

One hundred and fifty patients with breast cancer who were operated on after NAC between January 2016 and March 2020 were retrospectively analyzed. Age, gender, tumor size, axilla lymph node status, histopathological type, molecular subtypes, nuclear grade, and neutrophil, lymphocyte, and platelet counts were obtained and assessed before NAC. Pathologic responses to NAC were obtained from the postoperative pathology reports. NLR was calculated by dividing the neutrophil count (n/μl) by the number of lymphocytes (n/μl), and PLR was obtained by dividing the number of platelets (n/μl) by the number of lymphocytes (n/μl). Guided by the literature, the cut-off value for PLR was determined as 150 [6]. The receiver operating characteristic (ROC) curve analysis and diagnostic screening tests were used to determine the cut-off point for NLR.

Pathological assessment

The expression of the estrogen receptor (ER) and progesterone receptor (PR) was determined by immunohistochemistry. Tumors with ER (+) and PR (+) were classified as luminal types A or B according to the Ki-67 proliferation index being < or ≥14. Human epidermal growth factor receptor 2 (HER-2) expression was determined by immunohistochemistry or fluorescence in situ hybridization (FISH) depending on the case. Molecular subtypes were divided into four groups as luminal A, luminal B, HER-2 enriched, and triple-negative. The chemotherapy response of the tumor was evaluated according to Sataloff's criteria. Pathological complete response was evaluated as the absence of invasive disease in the breast and/or axilla (ypT0 and ypN0).

Systemic therapy

The patients were given cyclophosphamide, 5 fluorouracil, anthracycline, and/or taxanes in the NAC regimen, and additionally, trastuzumab was given to the group with HER-2 expression.

Statistical analysis

The Number Cruncher Statistical System (NCSS) 2007 (NCSS LLC, Kaysville, Utah, USA) was used for the statistical analysis. Descriptive statistical methods (mean, standard deviation, median, frequency, ratio, minimum, and maximum) were used when evaluating the study data. The suitability of quantitative data to normal distribution was tested by the Kolmogorov-Smirnov and Shapiro-Wilk tests and graphical evaluations. Student's t-test was used for comparing paired groups for normally distributed quantitative data, and the Mann-Whitney U test was used for the paired group comparisons of non-normally distributed data. In the comparison of qualitative data, the Pearson Chi-square and Fisher’s exact tests were used. Diagnostic screening tests (sensitivity, specificity, positive predictive value, and negative predictive value) and the ROC curve analysis were used to determine the cut-off value for NLR. Significance was evaluated at the level of p<0.05.

## Results

A total of 150 female patients with an average age of 45.6±10.7 years were operated on after NAC. Histopathologically, 75.3% of the patients had invasive ductal carcinoma. Nuclear grade 3 was reported in 68% of patients. According to molecular subtypes, triple-negative and HER-2 enriched tumors consisted 45.4%, whereas luminal types 54.6% of patients. Ki-67 proliferation index was greater than 14 in 84.7% of patients. Biopsy proven axillary lymph node metastasis was detected in 80% of the cases prior to NAC. After NAC, pCR in breast tissue alone, axillary nodes alone and both breast tissue and axillary nodes were determined in 42.7%, 44%, and 34.7% of the patients respectively (Table 1).

**Table 1:** Clinico-pathological features of patients (n=150) and pCR to NAC NAC: neoadjuvant chemotherapy; HER-2: human epidermal growth factor receptor 2; pCR: pathological complete response *Numbers in parentheses are percentages

Age		45.6±10.7 (range 18-79)	
Tumor size (mm)		33.14 (range 8-95)	
Histopathological types	Invasive ductal	113 (75.3)*	
	Invasive lobular	13 (8.7)	
	Other	24 (16)	
Ki67	<14	23 (15.3)	
	≥14	127 (84.7)	
Nuclear grade	1	9 (6)	
	2	39 (26)	
	3	102 (68)	
Molecular subtypes	Luminal A	29 (19.3)	
	Luminal B	53 (35.3)	
	Triple negative	24 (16.1)	
	HER-2 enriched	44 (29.3)	
Lymph nodes prior to NAC	Positive, metastatic		120 (80)
pCR	Breast + axilla	52 (34.7)	
	Breast	64 (42.7)	
	Axilla	66 (44)	

The ROC curve analysis and diagnostic screening tests were used to determine the cut-off value for NLR that was found to be 2.24. When the cases were evaluated according to their molecular subtypes, higher rates of low PLR (68.2%) and low NLR (63.6%) were found in HER-2-positive patients (Table 2).

**Table 2:** PLR and NLR levels according to molecular subtypes PLR: platelet-lymphocyte ratio; NLR: neutrophil-lymphocyte ratio; HER-2: human epidermal growth factor receptor 2 *Numbers in parentheses are percentages

	PLR Low (<150) High (≥150)			NLR Low (<2.24) High (≥2.24)		
Molecular subtypes	Low	High	p	Low	High	p
Luminal A (n=29)	17 (58.6)*	12 (41.4)	0.538	16 (55.2)	13 (44.8)	0.735
Luminal B (n=53)	29 (54.7)	24 (45.3)		28 (52.8)	25 (47.2)	
Triple negative (n=24)	13 (54.2)	11 (45.8)		13 (54.2)	11 (45.8)	
HER-2 enriched (n=44)	30 (68.2)	14 (31.8)		28 (63.6)	16 (36.4)	
Total (n=150)	89 (59.3)	61 (40.7)		85 (56.7)	65 (43.3)	

The relationship between molecular subtypes and pCR

The pCR rate was higher in patients with HER-2 and TN groups than with luminal subtypes (p<0.0001). When the pCR rate is evaluated according to breast and axilla separately, HER-2-positive patients had the best outcome ((p<0.0001)

The relationship between the PLR and NLR levels and pCR

The PLR ​​value was below 150 in 59.3% of the cases. The breast and axilla, breast alone and axilla alone pCR rates were significantly higher in the group with low PLR (p=0.013, p=0.018, and p=0.009, respectively). The NLR ​​value was below 2.24 in 56.7% of the cases (Table 3).

**Table 3:** pCR according to the PLR and NLR levels pCR: pathological complete response, PLR: platelet-lymphocyte ratio, NLR: neutrophil-lymphocyte ratio *Numbers in parentheses are percentages

	PLR Low (< 150) High (≥150)			NLR Low (< 2.24) High (≥ 2.24)		
Total (n=150)	Low (n=89)	High (n=61)	p	Low (n=85)	High (n=65)	p
Breast + axilla pCR (n=52)	38 (42.7)*	14 (23.0)	0.013	32 (37.6)	20 (30.8)	0.380
Breast pCR (n=64)	45 (50.6)	19 (31.1)	0.018	38 (44.7)	26 (40)	0.564
Axilla pCR (n=66)	47 (52.8)	19 (31.1)	0.009	43 (50.6)	23 (35.4)	0.063

The relationship between the PLR and NLR levels and pCR according to molecular subtypes

Despite pCR differences not being significant between patients with low or high PLR and NLR according to molecular subtypes, the best results were obtained in patients with low PLR and NLR, especially in the HER-2 group with low PLR and NLR (Tables 4, 5). 

**Table 4:** Pathological complete response according to molecular subtypes and PLR (low <150 and high ≥150) levels pCR: pathological complete response; PLR: platelet-lymphocyte ratio; HER-2: human epidermal growth factor receptor 2 *Numbers in parentheses are percentages

Molecular subtypes	PLR		Breast and Axilla pCR		p	Breast pCR			p		Axilla pCR		p	
														Luminal A (n=29)	Low (n=17)		0		^-^	1 (5.9)			1.0		1 (5.9)		1.0	
High (n=12)		0		1 (8.3)			0 (0)																				
Luminal B (n=53)	Low (n=29)		9 (31.0)*		0.226	12 (41.4)			0.111		13 (44.8)		0.394	
	High (n=24)		4 (16.7)			5 (20.8)					8 (33.3)			
Triple negative (n=24)	Low (n=13)		5 (38.5)		0.386	7 (53.8)			0.240		6 (46.2)		0.423	
	High (n=11)		2 (18.2)			3 (27.2)					3 (27.3)			
HER-2 (n=44)		Low (n=30)		24 (80)	0.152		25 (83.3)	0.434		27 (90)		0.019		
		High (n=14)		8 (57.1)			10 (71.4)			8 (57.1)				

**Table 5:** pCR according to molecular subtypes and NLR (low <2.24 and high ≥ 2.24) levels pCR: pathological complete response; NLR: neutrophil-lymphocyte ratio; HER-2: human epidermal growth factor receptor 2 *Numbers in parentheses are percentages.

Molecular subtypes	NLR	Breast and Axilla pCR	p	Breast pCR	p	Axilla pCR	p
Luminal A (n=29)	Low (n=16)	0		0	-	1 (6.3)	1.0
	High (n=13)	0		0		0 (0)	
Luminal B (n=53)	Low (n=28)	7 (25)*	0.933	7 (25)	0.933	13 (46.4)	0.284
	High (n=25)	6 (24)		6 (24)		8 (32)	
Triple negative (n=24)	Low (n=13)	4 (30.8)	1.0	4 (30.8)	1.0	5 (38.5)	1.0
	High (n=11)	3 (27.3)		3 (27.3)		4 (36.4)	
HER-2 (n=44)	Low (n=28)	21 (75)	0.732	21 (75)	0.732	24 (85.7)	0.25
	High (n=16)	11 (68.8)		11 (68.7)		11 (68.8)	

We analyzed the predictive value of PLR and NLR combinations for pCR. Patients with both low PLR and low NLR profiles had the highest pCR, between 30% and 75%, according to molecular subtypes (Tables 6, 7).

**Table 6:** Relationship of low PLR (<150) and low NLR (<2.24) with pCR according to molecular subtypes pCR: pathological complete response; NLR: neutrophil-to-lymphocyte ratio; HER-2: human epidermal growth factor receptor 2 *Numbers in parentheses are percentages

Molecular subtypes	Low PLR (n=89)	pCR	Low NLR (n=85)	pCR	Low PLR & NLR (n=69)	pCR	p	p
Luminal A (n=29)	17	0	16	0	11	0	-	0.0002
Luminal B (n=53)	29	9 (31)*	28	7 (25)	23	7 (30.4)	0.19	
Triple negative (n=24)	13	5 (38.5)	13	4 (30.8)	11	5 (45.5)	1.0	
HER-2 (n=44)	30	24 (80)	28	21 (75)	24	18 (75)	0.0003	
Total	89	38 (42.7)	85	32 (37.6)	69	30 (43.5)		

**Table 7:** Relationship of NLR/PLR combination with pCR pCR: pathological complete response; PLR: platelet-to-lymphocyte ratio; NLR: neutrophil-to-lymphocyte ratio *Numbers in parentheses are percentages.

NLR / PLR	Patients (n=150)	pCR (n=52)	p
High / High	45 (30)*	9 (20) (17.3)	
Low / High	16 (11)	5 (31.2) (9.6)	0.358
High / Low	20 (13)	11 (55) (21.2)	0.004
Low / Low	69 (46)	27 (39.1) (51.9)	0.031
Total	150 (100)	52 (100)	

## Discussion

Breast cancer is a heterogeneous disease. Perou and Sorlie showed that breast cancer could be sub-classified into different subtypes through the molecular analysis of patients according to gene expression profiling [1]. To date, decision-making for individual patients has been based on several factors, including tumor morphology and grade classification, tumor size, presence of lymph node metastases, and molecular subtype. Neoadjuvant chemotherapy has become the standard treatment in locally advanced breast cancer. Starting treatment with NAC increases the chances of breast and axilla-sparing surgery and reduces the risk of postoperative recurrence [2]. Studies have shown that response to NAC is a strong prognostic factor to predict long-term clinical outcomes, such as disease-free and overall survival, but there is still no definitive marker to predict patients that will respond well to treatment [3,4]. We aimed to study on prediction for better and higher pCR to NAC, using easy detectable inflammatory markers associated with molecular subtypes to predict pCR to NAC. 

Clinico-pathological features (Ki67 level, nuclear grade, molecular subtypes, and axillary status) of our candidate patients for NAC showed that the majority of them had relatively aggressive tumors with regional metastasis. In all cohort, overall pCR reaching 35% was an acceptable, considerable result for in vivo response to chemotherapy and favorable prognosis in breast cancer cases. In the literature, the pCR rate has been reported between 20% and 40%, which is consistent with our results [5]. In previous studies, the highest pCR rate was observed in HER-2-enriched tumors, followed by triple-negative and luminal type tumors [6,7]. In this study, while luminal A tumors had no pCR, pCR was detected in luminal B tumors at 24.5%, HER-2-enriched tumors at 72.7%, and triple-negative tumors at 29.1%.

Platelet-lymphocyte ratio according to molecular subtypes

In our series, we did not observe pCR in patients with luminal A tumors. We can comment that this type of tumors has low sensitivity to chemotherapy. Based on pathological response exclusion of luminal A tumor from NAC planning can be considered for a better outcome. On the other hand, TN and HER-2 tumors had much better pCR than luminal subtypes. More aggressive subtypes had a higher response rate to chemotherapy that they deserve more attention deciding upfront chemotherapy.

Platelet-lymphocyte ratio according to inflammatory markers (PLR and NLR)

The majority of patients had low ratios of inflammatory markers, especially patients with HER-2 subtype. Based on pCR rates, we can comment that patients with low PLR and low NLR had better pathological response to NAC than patients with higher ratios. Independent of molecular subtypes, low PLR, and low NLR appeared good predicting parameters for pCR in breast cancer cases undergoing to NAC. 

Platelet-lymphocyte ratio according to molecular subtypes associated with inflammatory markers (PLR and NLR)

It is well known that systemic inflammation plays an important role in tumor progression. Cancer cells secrete chemokines and cytokines, which allow leukocytes to migrate to the tumor. Neutrophils release substances that initiate angiogenesis, such as proangiogenic chemokines and vascular endothelial growth factor. Many studies report that increased inflammatory markers, such as NLR and PLR indicate a poor prognosis in patients with different solid organ malignancies, and NLR and PLR may be related to chemosensitivity [7-17].

The role of the host immune response in tumor development and cancer progression in breast cancer has been proven [18]. It is considered that NLR and PLR can be used as inflammatory markers due to the presence of an inflammatory response in cancer patients, and neutrophils, lymphocytes, and platelets play an important role in this inflammatory response. The effects of platelets on cancer cells are not well known, but some studies have reported that by covering the tumor cells, they prevent tumor cells from being recognized by the natural killer cells of the immune system [19]. Platelet-derived growth factors have been found to contribute to tumor growth, invasion, and metastasis when released by cancer cells.

In a previous study accepting the cut-off value of PLR as ≥292, a high PLR level was found to be related to lymph node metastasis and tumor grade, but no correlation was found between PLR and other clinicopathological features [12]. On the other hand, in a study by Koh et al., a high PLR level (cut-off:>215) was found to be related to age (>50 years) and tumor size (>5 cm), while it was not associated with lymph node metastasis and tumor grade [11]. Cuello Lopez et al. did not report a correlation between the PLR level and clinicopathological features (age, postmenopausal status, tumor grade, tumor size, lymph node metastasis, and disease stage) [6]. In the current study, there was no statistically significant relationship between the PLR and NLR levels and age, tumor size, tumor grade, histopathological type, lymph node metastasis, Ki-67 levels, and molecular subtype. Considering all these studies, the reason for the different results in the relationship between the PLR-NLR levels and clinicopathological features may be due to tumor heterogenecity, different cut-off values, low number of patients in studies, and differences in samples.

The importance of PLR and NLR in predicting response to treatment has been investigated in cases where treatment is started with chemotherapy. In the literature, a high pCR rate was found in patients with low PLR independent of molecular subtypes [6,14,20]. In the current study, there was a statistically significant difference in the pCR rate according to the PLR levels. The rate of pCR was higher in patients with low PLR (Table 4).To the best of our knowledge, there is no study in the literature evaluating the efficacy of the PLR level in predicting pathological response in the breast and axilla separately. In our study, the rates of pCR in the breast and axilla were higher in the group with PLR ​​<150. In the low PLR group, the pCR rate was increased independent of molecular subtypes. When the breast and axillary pCR values were separately evaluated according to molecular subtypes, in the HER-2-enriched group, the rate of pCR in the axilla was higher among the patients with PLR <150, but no significant difference was observed in the remaining comparisons. This information may be of clinical importance in the prediction of the axillary response after NAC and the decision to perform axillary surgery.

In recent years, NLR has been used as a systematic marker for inflammation, and its prognostic significance has been evaluated in several studies [7,15]. While Chen et al. and Asano et al. found a higher rate of pCR in patients with low NLR levels, no relationship was found between NLR and pCR in studies conducted by Eryilmaz et al. and Suppan et al [21-24]. In a meta-analysis, the relationship between NLR and pCR was investigated in many types of solid cancer, and a significant relationship was reported between the NLR levels and pCR in bladder and rectum cancer whereas there was no relationship between NLR levels and pCR in breast cancer [25]. In another meta-analysis consisting of 11 studies, although there was a statistically significant relationship between a high NLR level and patient response to NAC, NLR had no effect on disease-free survival and overall survival [26]. In this study, we did not find any relationship between the NLR level and pCR. Graziano et al. and Kim et al. investigated the value of the combination of NLR and PLR in predicting response to NAC. The combination of NLR and PLR was found to be a prognostic factor in patients treated with NAC, and a combination of low NLR and PLR was shown as a potential predictor of a better NAC response [27,28]. In our study, response to NAC was observed to be better in the group with low levels of both PLR and NLR.

In the literature, different cut-off values ​​are used for NLR and PLR. In some studies, cut-off values were determined using the ROC curve analysis while in others, predetermined cut-off values ​​were used [7,20,28]. The absence of a standard cut-off value may explain the differences in the results of the studies. There are also studies that determined different cut-off values ​​for different ethnicities; thus, racial and individual characteristics are considered to result in differences. In the current study, as in previous research, the cut-off value was accepted as 150 for PLR, and it was determined using the ROC curve analysis for NLR.

## Conclusions

Since it is very common to start breast cancer treatment with NAC, it is important to predict cases that will respond well to treatment. Neutrophil-lymphocyte ratio and PLR values ​​are inexpensive, repeatable, and easily accessible parameters that can be calculated by a routine blood count analysis. Multicenter studies using standard values ​​are needed to predict the chemotherapy response of NLR and PLR and to use them routinely as prognostic markers in clinical practice.
